# Neuropeptide Y innervation during fracture healing and remodeling: A study of angulated tibial fractures in the rat

**DOI:** 10.3109/17453674.2010.504609

**Published:** 2010-10-08

**Authors:** Hua Long, Mahmood Ahmed, Paul Ackermann, André Stark, Jian Li

**Affiliations:** Section of Orthopaedics, Department of Molecular Medicine and Surgery, Karolinska Institutet, StockholmSweden

## Abstract

**Background and purpose:**

Autonomic neuropeptide Y (NPY) is involved in local bone remodeling via the central nervous system. However, the role of peripheral neuronal NPY in fracture healing is not known. We investigated the relationship between bone healing and side-specific occurrence of NPY in angular and straight fractures.

**Methods:**

Tibial fractures in Sprague-Dawley rats were fixed with intramedullary pins in straight alignment and anterior angulation. The samples were analyzed by radiography, histology, and immunohistochemistry (IHC) between 3 and 56 days postfracture.

**Results:**

In the angular fractures, radiography and histology showed a 3.5-fold increase in callus thickness on the concave side compared to the convex side at day 21, whereas a 0.2-fold reduction in callus thickness was seen on the convex side between days 21 and 56. IHC showed regenerating NPY fibers in the callus and woven bone in both fractures at day 7. In angular fractures, a 5-fold increase in NPY fibers was observed on the concave side compared to the convex side at 7 days, whereas a 6-fold increase in NPY fibers was seen on the convex side between 21 and 56 days; only a 0.1-fold increase in NPY fibers was seen on the concave side during the same time period. In straight fractures, similar bony and neuronal changes were observed on both sides.

**Interpretation:**

The increase in NPY innervation on the convex side appears to correlate with the loss of callus thickness on the same side in angular fractures. Our results highlight the probable function of the peripheral NPY system in local bone remodeling.

The ability of bone to maintain its structures and properties or to remodel after trauma under different conditions is well known, but the underlying neurobiological mechanisms are poorly understood. The bone has a rich supply of nerve fibers, which can be classified as belonging to the sensory, autonomic, and opioid nervous systems according to the phenotype of the neurotransmitters (Bjurholm et al. 1988, Hill et al. 1991). This strongly supports the peripheral role of neuropeptides in bone physiology. Recently, the role of neural signaling by the central nervous system in bone remodeling through various pathways has been given some attention. One such pathway is the neuropeptide Y (NPY) system. NPY, a 36 amino acid peptide, belongs to a class of peptides that includes pancreatic polypeptide (PP) and peptide-YY (PYY), which are expressed in the central and peripheral autonomic nervous system. This system signals through 5 distinct receptors (Y1, Y2, Y4, Y5, and Y6) expressed in various tissues. NPY has several functions: among these are effects on energy homeostasis, food intake, immunity, and the cardiovascular system (Lin et al. 2004). Recently, an important role of NPY in bone homeostasis has been identified through its effect on Y2 and Y1 receptors: a study in mice with hypothalamic deletion of Y2 receptors showed a generalized increase in bone formation (Baldock et al. 2002). In contrast to this central action of Y2 receptors, the presence of Y1 receptors on osteoblasts suggests a direct local effect on these cells (Lundberg et al. 2007). It has been shown that mice deficient in Y1 receptors have a high bone mass (Baldock et al. 2007). Whether it occurs through NPY-ergic pathways emanating from the hypothalamus or through skeletal NPY innervation has not been determined.

In the bone, NPY-immunoreactive fibers have been identified in areas of high osteogenic potential such as bone marrow and the periosteum (Bjurholm et al. 1988, Hill et al. 1991). These nerve fibers were found to be arranged as networks, mainly around blood vessels. Experimental studies in vivo have shown that NPY acts as a potent vasoconstrictor of blood vessels in bone (Lindblad et al. 1994), but its role in local bone turnover is not clear. Moreover, under conditions of trauma it is not known whether NPY participates in different biological events such as inflammation, cell proliferation, angiogenesis, vasoregulation, and callus formation.

Recently, we reported a rat model of angulated tibial fracture that showed side-specific changes in bone formation on the concave side and bone resorption on the convex side of deformities in the same bone (Li et al. 2004, 2007). The process of bone formation/resorption in this animal model, leading to bone healing and remodeling, might be affected by peripheral expression of NPY because of its role in skeletal homeostasis. We therefore assessed the temporal and side-specific expression of NPY at the fracture site during healing and remodeling by morphological and semiquantitative analysis using immunohistochemistry.

## Materials and methods

44 male Sprague-Dawley rats with a mean weight of 200 g were housed 3 per cage with free access to standard rat chow and water under controlled temperature and a 12-hour light/dark cycle. All animal experiments were performed with approval from the Ethics Committee for Animal Research, Stockholm North. The rats were allocated to 3 groups as follows: 20 rats with a tibial fracture in anterior angulation (group A); 20 rats with a tibial fracture in straight alignment (group B); and 4 control rats with intact tibia (group C).

### Surgery

The rats were anesthetized with an intraperitoneal (i.p.) injection of fentanyl-fluanisone (Hypnorm, 0.5 mL/kg). The right tibia was first weakened at the mid-diaphysis by percutaneous drilling with an 18-G needle. A small anteromedial skin incision was made proximal to the tibial tubercle and a 17-G cannula needle was inserted through the proximal metaphyseal cortex into the medullary canal as described previously (Li et al. 2004). The tibia was then fractured by manual 3-point bending and fixed in either 40° anterior angulation or straight alignment, which in both cases included the normal 13° anatomical curve of the diaphysis.

### Radiography

**Figure 1.**
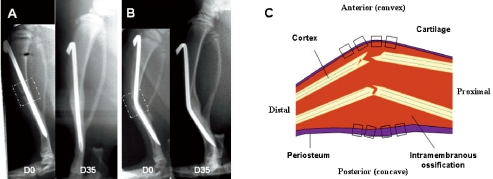
Lateral view of tibia at day 0 and day 35 postfracture, fixed in straight alignment (A) and anterior angulation (B). The broken squares in the X-ray images (panels A and B) depict the dissected sample for microscopic analysis of longitudinal sections shown in the schematic illustration (C). The small dotted squares in the drawing of the dissected sample represent the 8 microscopic fields studied in each tissue section for morphological and semi-quantitative analysis of NPY immunoreactivity. D = day.

The progress of fracture healing was monitored on lateral radiographs under Hypnorm anesthesia on day 0, 7, 21, 35, and 56 after fracture (Figure 1A and B). The radiographic set-up was adjusted to result in size enhancement of the image by 33%. Callus size was then measured manually on a 300% magnified printout. The total callus size was assessed by measuring the transverse diameter (in mm) of the callus and the thickness of cortical bone anterior and posterior to the nail at the level of the apex of the angulated needle.

### Sampling

The rats were anesthetized with sodium pentobarbitone (60 mg/kg, i.p.). 4 rats in each fracture group were killed on days 3, 7, 21, 35, and 56, while the 4 control rats with intact tibias were killed on day 21. In vivo intra-aortic perfusion was carried out with phosphate-buffered saline (PBS), followed by fixation with Zamboni's buffered 4% paraformaldehyde solution containing 0.2% picric acid in 0.2 M Sörensen phosphate buffer, pH 7.2. The right tibia was dissected and then demineralized at room temperature in a solution containing 7% AlCl3, 5% formic acid, and 8.5% HCl for 12 h. The samples were soaked in 20% sucrose for at least 2 days. Each demineralized tibia was divided sagittally in two halves and 2-cm long samples of the medial half of the tibial diaphysis, which included the proximal, middle, and distal parts of the healing fracture, were collected for analysis. Subsequently, the 2-cm long samples were sectioned at a thickness of 15 μm using a cryostat. Thus, the regions undergoing endochondral and intramembranous ossification were all included in the same section.

### Immunohistochemistry

Serial sections were numbered consecutively from the middle to the medial aspect. Two-interval sections, i.e. one close to the middle part and another close to the medial part of the tibia at each time point, were chosen for H & E staining and for immunostaining according to the biotin-avidin system. Thus, the sections were hydrated in 0.01 M PBS for 10 min and then incubated with 10% normal goat serum at room temperature for 30 min. Subsequently, the sections were incubated at room temperature overnight in a humid atmosphere with polyclonal antibody to NPY (1:5,000; Peninsula Laboratories, San Carlos, CA). After rinsing in PBS (2 × 5 min), the sections were incubated with biotinylated goat anti-rabbit antibody (1:250; Vector Laboratories, Burlingame, CA) for 40 min. Finally, fluorochrome Cy3-conjugated avidin (1:5,000; Amersham) was used for visualization of the immune reaction. Control staining was performed by omitting the primary antibody. Addition of 50 μL of peptide to the corresponding antiserum before application to tissue sections served as another control.

For double staining, after completing the staining step for NPY using fluorochrome Cy2-conjugated avidin for visualization of the immunoreaction, the sections were incubated for 15 min with avidin-blocking solution followed by biotin-blocking solution. The sections were then incubated with goat anti-mouse monoclonal antibody to GAP-43 (1:2,000), a marker of axon growth, neuronal development, and nerve regeneration, and mouse polyclonal antibody to PGP 9.5 (1:10,000; both antibodies from Boehringer Mannheim Biochemicals, Germany), the marker for mature nerve fibers. Then the sections were incubated with biotinylated anti-mouse antibodies (1:250; Vector Laboratories) for 40 min. The fluorochrome Cy2-conjugated avidin (1:1000; Amersham) was used for visualization of the immunoreaction. An epifluorescence microscope was used for analysis (20× objective).

### Semiquantification

The nerve fiber density in and adjacent to the fracture area during healing and modeling was quantified by computerized image analysis as previously described (Li et al. 2007). Briefly, 2 consecutive tissue sections per rat were included. In each tissue section, 4 fields (20×) of each were selected in a standardized manner from the concave and convex sides, for comparison and quantification (Figure 1C). For quantification by computerized image analysis, images of the tissue sections were captured by a video camera attached to the immunofluorescence microscope and then saved in a computer. The fields selected were analyzed using Easy Analysis software (Bergström Instruments, Stockholm, Sweden). A standard lower and upper threshold of fluorescence intensity was consistently applied for positively stained nerve fibers. The results were expressed as the nerve fiber immunofluorescent area in relation to the total area of each microscopic field. Since there were 2 tissue sections in each rat, 8 microscopic fields representing the convex side and 8 other fields representing the concave side were analyzed. The mean value (the percentage of neuronal immunofluorescenc area) based on 8 microscopic fields was determined in 4 rats to obtain a measure of fiber density for comparison of the concave and convex sides of each tibia.

### Statistics

For semiquantitative analysis of neuronal NPY-positive immunofluorescence area, a non-parametric approach was used. In each experimental group, the statistical analysis was based on the mean of the values from 4 rats at each time point. Since our data was skewed, the results are presented as mean, median, and range (minimum and maximum). Mean values were used to calculate the fold differences in NPY immunoreactivity whereas median values were used to obtain the statistically significant differences between the sides. To compare the differences between the convex and concave sides within the same group, the Wilcoxon test was used. A p-value of < 0.05 was considered statistically significant.

## Results

### Radiography

Radiographic analysis showed a statistically significant increase in total callus formation from day 0 to day 21 postfracture (inflammatory and proliferative phases) in both fracture groups. When compared to the bone thickness of each side at day 0, the angular fracture exhibited a 3.5- fold increase in callus thickness on the concave side, whereas only a 0.2-fold increase in callus thickness was observed on the convex side at day 21 (p = 0.03). During the remodeling phase, i.e. between day 21 and day 56, a 0.2-fold reduction in callus thickness was observed on the convex side (p = 0.05), while the callus thickness remained almost the same on the concave side, as compared to day 21. In the straight fracture, an almost equal increase in callus thickness was observed on the concave side (1.9-fold) and on the convex side (1.1-fold), reaching a peak at day 21. It then decreased gradually, in a similar way on both sides.

### Histology

**Figure 2.**
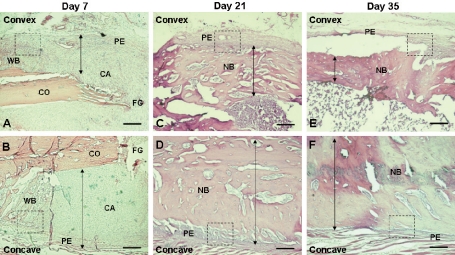
Hematoxylin and eosin (H & E) staining (A-F) showing changes in bone thickness on the convex (anterior) side (A, C, and E) and the concave (posterior) side (B, D, and F) of the angulated fracture at day 7 (A and B), 21 (C and D), and 35 (E and F) after fracture. The arrows depict the thickness of woven bone or new bone, showing intensive bone formation on the concave side at early phases of fracture healing (days 7 and 21) and intense reduction of bone thickness on the convex side at a later phase of fracture healing (day 35). The small dotted square in each panel depicts the observed area of NPY immunostaining (20×) shown specifically in Figures 4 and 5. 2× objective. Bar represents 1,000 μm. CA: cartilaginous callus; CO: cortical bone; FG: fracture gap; NB: new bone; PE: periosteum; WB: woven bone.

The longitudinal sections of tibia were stained with H & E at 7, 21, and 35 days postfracture in both fracture groups (Figure 2). In the straight fracture, a similar histological picture depicting different phases of bone repair was seen on the two sides of the fracture. Thus, there were equal amounts of new woven bone and callus formation at day 7, complete thickened cortical bridging at day 21, and reduction in cortical size with normal bone architecture at day 35. However, in the angular fracture a different histological picture was seen, with disparity in bone repair between the concave and the convex sides. The increase in the size of new woven bone and callus formation was greater on the concave side than on the convex side at day 7 (Figure 2A and B). At day 21, cortical bridging was complete with periosteal hypertrophy and intense new bone formation on the concave side, whereas these changes were much less apparent on the convex side (Figure 2C and D). At day 35, a further reduction in cortical thickness was observed on the convex side whereas it remained the same on the concave side, representing normal bone architecture (Figure 2E and F). The morphological changes representing callus thickness between the concave and convex sides were based on observations in 4 rats at each time point (Figure 2A and F).

### Distribution of NPY nerve fibers in fractures

NPY-positive nerve fibers were identified in the intact tibia and at the fracture site in both straight and angular fractures at all time points of the study.

**Figure 3.**
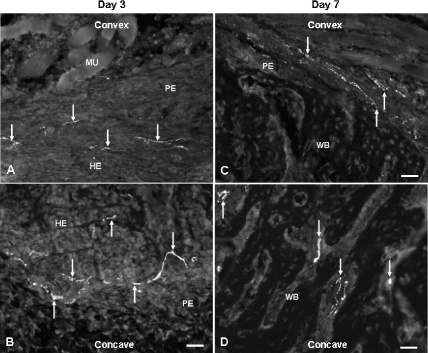
Fluorescence photomicrographs showing site-specific occurrence of NPY fibers on the convex (A and C) and concave (B and D) sides of angulation fractures at days 3 (A and B) and 7 (C and D) after fracture. 20× objective. Bar represents 50 μm. Arrows show NPY fibers. HE: hematoma; MU: muscle; PE: periosteum; WB: woven bone.

**Figure 4.**
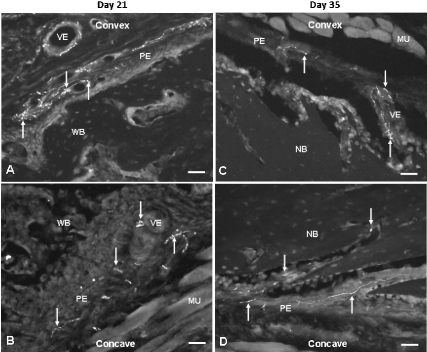
Fluorescence photomicrographs showing site-specific occurrence of NPY fibers on the convex (A and C) and concave (B and D) sides of angulation fractures at days 21 (A and B) and 35 (C and D) after fracture. 20× objective. Bar represents 50 μm. Arrows show NPY fibers. NB: new bone; MU: muscle; PE: periosteum; VE: vessel; WB: woven bone.

In angulated fractures, ingrowth of nerve fibers containing NPY was already observed at day 3 postfracture in the fracture hematoma arranged as non-vascular nerve fibers (Figure 3A and B). At day 7, an increased number of NPY-positive fibers was found on the concave side compared to the convex side. These nerve fibers were seen as fibers sprouting into the fibrocartilage callus from the deep layers of the periosteum. Many fibers were seen close to the chondroid cells, and they had entered the new-woven bone (Figure 3C and D). Between days 21 and 35, NPY-positive fibers were mainly observed as networks in and around the walls of blood vessels, both in the deeper layers of hypotrophic periosteum and in the new bone on the concave side, whereas few vascular fibers entering into new bone were found on the convex side (Figure 4A and B). At day 56 postfracture, during the remodeling, most of the NPY-positive fibers were seen retracted from the fracture site and were present as vascular fibers in the periosteum.

In straight fractures, the occurrence and distribution of NPY-positive fibers over the time period was similar to that seen in the angular fracture, with one notable difference. An increased number of NPY-positive fibers with similar amount were seen penetrating the woven bone on both sides of the fracture at day 7. No side-specific differences in expression were found over time.

**Figure 5.**
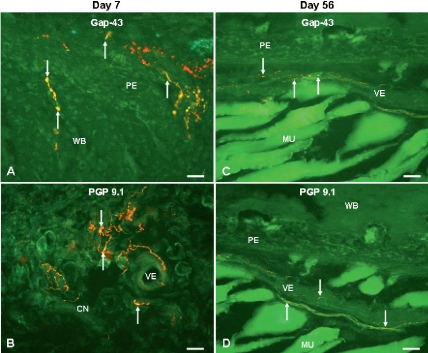
Fluorescence photomicrographs showing double staining of NPY and GAP-43 (A and C) or PGP 9.5 (B and D) at the fracture site in angulation fractures at days 7 (A and B) and 56 (C and D) after fracture. Co-localization of NPY (red) with GAP-43 (green) and with PGP 9.5 (green), resulting in yellow color, as seen in the woven bone and hypertrophic periosteum (A) and connective tissue (B) at day 7 and vessels (C and D) at day 56. 20× objective. Bar represents 50 μm. Arrows show co-localization of NPY with GAP-43 or with PGP 9.5. CN: connective tissue; MU: muscle; PE: periosteum; VE: vessel; WB: woven bone.

Double-staining experiments showed that there was co-expression of GAP 43, a marker of regenerating nerve fibers, with NPY-positive fibers at the fracture site in both groups at all time points. The co-localization of GAP 43 with NPY was more intense between days 3 and 21 postfracture than that at the later time periods (Figure 5A and C). Double-staining studies of PGP 9.5, a neuronal marker of mature nerve fibers and NPY showed a co-localization that was less obvious at days 3–21, but which became more pronounced at days 35–56 postfracture (Figure 5B and D).

### Semiquantitative analysis of NPY innervation

In general, there was a statistically significant increase in the number of nerve fibers expressing NPY at the fracture site in both straight and angular fractures at days 7, 21, and 35 postfracture as compared to intact tibia (Table).

**Table:** Changes of NPY immunoreactivity in straight and angulated fractures

	Straight fracture							Angulated fracture						
	Posterior side			Anterior side				Posterior (concave) side			Anterior (convex) side			
Day	A	B	C	A	B	C	D	A	B	C	A	B	C	D
3	–0.07	–0.15	–0.28–0.29	–0.17	–0.26	–0.33–0.17	0.1	0.11	–0.16	–0.47–1.21	–0.18	–0.38	–0.60–0.70	0.4
7	0.45	0.39	0.09–0.92	0.22	0.10	–0.16–0.85	0.5	0.50	0.53	0.35–0.61	–0.51	–0.53	–0.62 to –0.35	0.04
21	0.44	0.44	–0.04–0.91	0.60	0.45	0.17–1.33	0.7	0.67	0.67	0.67–0.67	0.86	0.66	0.54–1.39	0.9
35	0.85	0.54	0.08–2.22	0.70	0.39	0.07–1.97	0.1	0.52	0.45	0.21–0.98	0.75	0.67	0.48–1.18	0.8
56	0.01	0.06	–0.40–0.34	–0.07	–0.21	–0.57–0.69	0.5	0.16	0.15	0.06–0.29	0.04	0.06	–0.06–0.10	0.1

The percentage changes of NPY immunoreactivity in both straight fracture and angulated fracture at each time point as compared to intact (control) bone. A Mean B Median C Range D P-value

In angular fractures, a 5-fold increase in NPY-immunoreactive fibers was seen on the concave side as compared to the convex side when comparison was made with the innervation of intact bone at day 7. Notably, a statistically significant (6-fold) increase in NPY-positive fibers was seen on the convex side between days 21 and 56, whereas the corresponding increase in NPY was only 0.1-fold on the concave side during the same time period, when compared to day 7 (Table). Interestingly, the peak in NPY-positive fibers was only seen on the convex side in the angular fractures at days 21 and 35. Although the amount of NPY-positive fibers was similar on the concave and convex sides in the angular fractures between days 21 and 56, an increase in NPY innervation on the convex side was observed, but it was not statistically significant.

In the straight fractures, a statistically significant increase in NPY-positive fibers was found both on the concave side (by a factor of 0.6) and on the convex side (by a factor of 0.4) when compared to intact tibia at day 7. The maximum increase in NPY-positive fibers (by a factor of 1) was observed on both sides in the straight fractures at day 35, the phase of bone remodeling. Later, the density of NPY fibers returned to the normal (intact) level on both sides of the fracture (Table).

## Discussion

Our study demonstrates temporal and side-specific changes in NPY innervation during the early and the late phase of angular fracture healing. The early increase in NPY innervation seen on the bone-forming concave side during the inflammatory phase may be a response to injury with the possible stimulatory effects on cell proliferation and angiogenesis leading to enhanced bone formation. The increased expression of NPY seen on the convex (bone resorption) side during the remodeling phase appears to coincide with the reduction in callus thickness. The later increase in NPY expression on the convex side may reflect the role of NPY in bone resorption, possibly through the Y1 receptor. Given the proposed central role of NPY in bone homeostasis, our results suggest that NPY may have a peripheral neuronal function in local bone turnover, especially during fracture healing and correction of angular deformity.

The early inflammatory phase of fracture healing lays the foundation for bone healing as inflammatory cells secrete a number of proinflammatory cytokines with bone induction properties. Accordingly, the cascade of reparative processes such as matrix synthesis, cell proliferation, angiogenesis, and neurogenesis is induced. Our results show an intense but similar pattern of nerve regeneration containing autonomic neuropeptide NPY in the fracture callus and woven bone on the two sides of a straight fracture (Figures 3 and 5). However, in an angular fracture this phenomenon of nerve regeneration was observed mainly on the concave side (Figure 3B and D) (Table). The regenerative nature of NPY immunoreactive fibers observed in the inflammatory phase of fracture healing was confirmed by identification of co-expression of GAP-43, a neuronal marker of nerve regeneration, in the NPY-positive fibers (Figure 5). It is presumed that increased delivery of NPY from the nerve endings during the inflammatory phase of fracture healing would exert a beneficial effect on bone repair by modulating various cellular events. A number of studies have suggested that NPY exerts a strong angiogenic effect, mainly on the Y2 receptor (Y2R) (Ekstrand et al. 2003, Lee et al. 2003). In vitro studies have shown that NPY promotes vessel sprouting and adhesion, migration, proliferation, and capillary tube formation in human endothelial cells (Zukowska-Grojec et al. 1998, Movafagh et al. 2006). Furthermore, it has been shown that vessel sprouting is reduced to 50% after deleting Y2R or by using antagonists of Y2R (Lee et al. 2003). In addition, a delay in skin wound healing and reduced neovascularization was observed in mice lacking Y2R (Ekstrand et al. 2003). It is known that early angiogenesis at the fracture site is one of the most important parameters influencing the healing process (Keramaris et al. 2008). Our study has shown early proliferation of nerve fibers containing NPY at the fracture site where increased callus formation is observed (concave side in angular fractures and on both sides in straight fractures), which possibly contributes to the healing process by stimulating angiogenesis (Figure 3 and 5). In addition to angiogenesis, recent studies have shown a possible role of NPY in neurogenesis. A potential role of NPY in the survival and the regenerative process of neurons was indicated by the dramatic increase in NPY expression in dorsal root ganglia after peripheral nerve injury (Wakisaka et al. 1991). Moreover, a possible proliferative effect of NPY on neuroblasts in different areas of the neonatal and adult brain has been shown to be mediated mainly through Y1R (Howell et al. 2003, 2005). In addition to the CNS, NPY has also been identified as a neurotrophic factor in the peripheral nervous system by showing its regenerative effect on olfactory epithelium (Hansel et al. 2001, Hökfelt et al. 2008). It has been shown that NPY acting through Y1R stimulates the proliferation of adult olfactory neuroblasts. Conversely, NPY-deficient mice were found to show a significant reduction in olfactory neuronal precursor cell proliferation (Hansel et al. 2001). Recently, it has been shown that increased release of NPY promotes the regeneration of damaged olfactory sensory neurons (Kanekar et al. 2009). Altogether, the role of NPY implicated in neuroangiogenesis might set up the initial framework for a satisfactory bone-healing process.

We have recently established a rat model of angular fracture of the tibia, which covers not only the healing process but also the subsequent bone remodeling (Li et al. 2004, 2007). In these studies, angulation gave a clear-cut difference in local bone turnover between the concave and convex sides of the fracture. We also found intense bone formation on the concave side whereas bone resorption predominated on the convex side, both sides thereby contributing to spontaneous correction of the angular deformity (Figure 1 and 2). Notably, our angular fracture model also permits sequential analysis of local mediators involved in bone formation and resorption within well-defined areas of the same bone at different stages of fracture healing. Thus, we have observed increased expression of the sensory neuropeptide CGRP on the concave side, where intense new bone formation occurred. On the convex side where bone resorption predominated, there was an increase in the occurrence of the bone resorption cytokine interleukin-1 (Li et al. 2004, 2007), as confirmed by others (Gordon et al. 2008).

In the present study a reduction in callus thickness during the remodeling phase which coincided with a 6-fold increase in NPY expression on the convex side, probably highlights the role of NPY in bone resorption. Even though there is no conclusive evidence to implicate NPY-mediated signaling in bone homeostasis, a number of studies have indicated the probable role of NPY in local bone turnover through the central and peripheral nervous system (Baldock et al. 2002, 2007, Lundberg et al. 2007). Recent studies analyzing germ line or conditional knockout mice lacking Y2R have revealed that the osteoblast activity is controlled by a central pathway through hypothalamus. These studies have shown an increased rate of bone mineralization and elevated bone mass due to increased osteoblast activity in Y2R knockout mice (Lundberg et al. 2007). However, the presence of neuronal NPY in high osteogenic areas such as the periosteum, epiphysis growth plate, and bone marrow may suggest a role of NPY in local bone turnover by a peripheral pathway (Bjurholm et al. 1988, Hill and Elde 1991). Recently, the Y1 receptor was reported to be present in human osteoblastic and osteosarcoma-derived cell lines (Lundberg et al. 2007), and treatment of wild-type mice with NPY caused reduction in bone mass and volume (Ducy et al. 2000). In addition, in vitro studies have demonstrated that NPY can inhibit the osteoblastic parathyroid hormone response through a receptor-receptor interaction (Bjurholm et al. 1992). A more direct effect of NPY on bone cells involved in bone remodeling has been reported. Not only the presence of Y1R on osteoblasts but also a generalized increase in bone formation on the cortical and cancellous surfaces of bone has been shown in Y1R-deleted mice (Lundberg et al. 2007). It can be assumed that the elevated levels of NPY seen in our study caused an overstimulation of Y1R on osteoblasts, leading to bone resorption on the convex side of the fracture.

In summary, we have demonstrated upregulation in neuronal expression of NPY during the early and late phases of angular fracture healing, thus highlighting a probable role of NPY in local bone remodeling. The findings of this study combined with those in previous reports suggest that NPY may play a role in the regulation of skeletal homeostasis, probably through Y1R. Therapeutic application of the NPY system has emerged as a promising area of research in clinical orthopedics. A useful approach in this regard would be the anti-ligand/anti-receptor strategy to prevent or minimize bone loss in a number of skeletal disorders such as tumor, trauma, and infection.
